# The Art Amateur

**Published:** 1894-08

**Authors:** 


### THE ART AMATEUR. July, 1894. Published by Montague Marks, 23 Union Square, New York. Price, 35 cents a number; $4 a year.

Contains two colored plates: 1, "The Shepherdess," by Chialiva, and 2, "Swallows and Reeds," by Helena Maguire. The frontispiece is "Portrait of a Man," engraved by Baude after the painting by Rembrandt. It gives interesting accounts of the "Salon" of the Champ de Mars, the "Salon" of the Champs Elyseés, and the Group Exhibition. The section called "Gallery and Studio" has sixteen illustrated articles. The supplement designs are a decoration for a bowl; Virginia Rail; fifth series of a set of game plates; Embroidery for a portière; Corner piece for embroidery; Initial letters; Carved hanging cabinet; Details for painted decoration for the legs of a table in a blue and white bed-room; Decorations for a mirror-frame, and frieze in the same, and designs for china painting. It gives pictures of artist studios and beautiful and unique designs for furnishing a bed-room in an inexpensive way. Amateurs in art should be subscribers to this valuable art monthly as it fully repays the money expended.

